# Cardiorespiratory physiology and swimming capacity of Atlantic salmon (*Salmo salar*) at cold temperatures

**DOI:** 10.1242/jeb.245990

**Published:** 2023-09-06

**Authors:** Emma S. Porter, A. Kurt Gamperl

**Affiliations:** Department of Ocean Sciences, Memorial University of Newfoundland and Labrador, St John's, NL, Canada, A1C 5S7

**Keywords:** Cold, Temperature, Acclimation, Cardiac function, Heart, Swimming, Oxygen extraction

## Abstract

We investigated how acclimation to 8, 4 and 1°C, and acute cooling from 8  to 1°C, affected the Atlantic salmon's aerobic and anaerobic metabolism, and cardiac function, during a critical swim speed (*U*_crit_) test. This study revealed several interesting temperature-dependent effects. First, while differences in resting heart rate (*f*_H_) between groups were predictable based on previous research (range ∼28–65 beats  min^−1^), with values for 1°C-acclimated fish slightly higher than those of acutely exposed conspecifics, the resting cardiac output (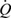) of 1°C-acclimated fish was much lower and compensated for by a higher resting blood oxygen extraction (*Ṁ*_O_2__/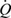). In contrast, the acutely exposed fish had a ∼2-fold greater resting stroke volume (*V*_S_) compared with that of the other groups. Second, increases in *f*_H_ (1.2- to 1.4-fold) contributed little to 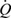 during the *U*_crit_ test, and the contributions of 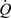 (*V*_S_) versus *Ṁ*_O_2__/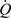 to aerobic scope (AS) were very different in the two groups tested at 1°C (1°C-acclimated and 8–1°C fish). Finally, *U*_crit_ was 2.08 and 1.69 body lengths (BL) s^−1^ in the 8 and 4°C-acclimated groups, but only 1.27 and 1.44 BL s^−1^ in the 1°C-acclimated and 8–1°C fish, respectively – this lower value in 1°C versus 8–1°C fish despite higher values for maximum metabolic rate and AS. These data: support recent studies which suggest that the capacity to increase *f*_H_ is constrained at low temperatures; show that cardiorespiratory function at cold temperatures, and its response to increased demands, depends on exposure duration; and suggest that AS does not constrain swimming capacity in salmon when chronically exposed to temperatures approaching their lower limit.

## Teaser

**Summary:** Cold temperatures constrain heart rate, metabolic capacity and swimming performance in salmon. However, acclimation and acute exposure to 1°C have different effects on the role played by stroke volume versus oxygen extraction in supporting oxygen consumption during exercise.

## INTRODUCTION

Temperature is considered the ‘master ecological factor’ with regard to the biology and physiology of aquatic ectotherms (Brett, 1971), and an extensive body of work on fishes has focused on thermal relationships across various levels of biological organization [see comprehensive reviews by Farrell (2009) and Eliason and Anttila (2017)]. Further, determining the optimal temperature range for key functional processes (i.e. growth, activity, swimming, reproduction, etc.) has proven to be extremely useful in efforts to manage ecologically and economically important species in the current era of accelerated climate change [i.e. increases in global average temperatures, and in the frequency and severity of challenging and/or harmful environmental events such storms, heat waves and drastic reductions in water oxygen content (hypoxia): Breitburg et al., 2018; Cooke et al., 2012; Horodysky et al., 2015; Killen et al., 2016; Little et al., 2020].

Average ocean temperatures are predicted to rise by 2–4°C by the end of this century (IPCC, 2018, 2022), and this has prompted in-depth research over the past several decades to understand the impacts of high water temperatures on the physiology of fish (e.g. Andreassen et al., 2022; Ekström et al., 2021; Ørsted et al., 2022). However, marine environments are also experiencing extreme reductions in temperatures at an increased rate, and for prolonged periods of time. These events are termed ‘cold shock’ or ‘winter chill’ (Reid et al., 2022; Szekeres et al., 2016; USGCRP, 2017), and only a few studies have investigated the cardiorespiratory responses of salmonids (temperate eurythermal fishes) to suboptimal or extremely cold temperatures (0–1°C). Specifically, with the exception of the recent work by Porter et al. (2022), the majority of *in vivo* research on salmonid cardiorespiratory function at cold temperatures has focused on: (i) hypertrophy versus hyperplasia of the myocardium (i.e. an increase in relative ventricular mass) (Driedzic et al., 1996; Klaiman et al., 2011); (ii) blood flow to the red (aerobic) and white (anaerobic) muscle (Taylor et al., 1996), or (iii) experiments where anaesthetized fish were given pharmacological agents (atropine and isoproterenol) and exposed to very rapid acute changes in temperature (e.g. Gilbert and Farrell, 2021). This is despite the fact that species such as the Atlantic salmon (*Salmo salar*) in the North Atlantic can experience temperatures as low as −0.5°C in the winter in the wild and at aquaculture cage-sites, and daily temperature variations of up to ∼10°C (Bjornsson et al., 2007; Strøm et al., 2019; Vadboncoeur et al., 2023).

Gamperl and Syme (2021) recently published data that strongly suggest that myocardial contraction/twitch kinetics greatly constrain maximal heart rate (*f*_H,max_) at cool or cold temperatures, and that this may require fish to elevate stroke volume (*V*_S_) to an equal or greater extent than *f*_H_ to meet demands for increased cardiac output (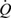). To test this hypothesis, and to examine how long- and short-term changes in temperature near the lower limits for this species affect cardiac function, metabolic physiology, blood oxygen carrying capacity and swimming performance, we acclimated Atlantic salmon to 8, 4 and 1°C, and exposed a group of 8°C-acclimated fish to an acute drop in temperature to 1°C. Our expectation was that an inability to increase *f*_H_ at cold temperatures would limit swimming performance and/or result in plastic responses in other physiological mechanisms that determine blood oxygen delivery to the tissues or the fish's overall metabolic capacity (i.e. *V*_S_, blood oxygen carrying capacity, tissue oxygen extraction (*Ṁ*_O_2__/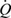) or anaerobic metabolism). These other mechanisms are key determinants of fish swimming capacity at ecologically relevant temperatures (Anttila et al., 2014; Claireaux et al., 2005; Eliason et al., 2013; Keen and Farrell, 1994; Steinhausen et al., 2008), and such knowledge is crucial if we are to better predict the full range of climate change impacts on fish populations, as well as develop strategies to ensure the health and welfare, and sustainable production, of fish cultured in northern temperate regions.

**List of abbreviations**

AS: aerobic scope (MMR−SMR)
AS_R_: ‘realistic’ aerobic scope (MMR−RMR)
CVP: central venous pressure
*f*_H_: heart rate
*f*_H,max_: maximum heart rate
FS: factorial scope
Hb: haemoglobin
Hct: haematocrit
*K*: condition factor
MCHC: mean cellular haemoglobin concentration
MMR: maximum metabolic rate
*Ṁ*_O_2__/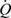
tissue oxygen extraction
*Ṁ*_O_2__: oxygen consumption
*P*_O_2__: partial pressure of oxygen
paCA: plasma-accessible carbonic anhydrase
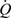
cardiac output
*Q*_10_: the fractional change in a rate over a 10°C range
RMR: routine metabolic rate
RVM: relative ventricular mass
SMR: standard metabolic rate
*U*_crit_: critical swimming speed
*V*_S_: stroke volume

## MATERIALS AND METHODS

This study was approved by the Animal Care Committee of Memorial University of Newfoundland and Labrador (protocol #21-01-KG). All procedures conducted on the salmon were performed in accordance with the Canadian Council on Animal Care's Guidelines on the ‘Care and Use of Fish in Research, Teaching and Testing’ (Canadian Council on Animal Care, 2005).

### Fish husbandry and rearing conditions

Juvenile mixed sex Atlantic salmon, *Salmo salar* Linnaeus 1758, from Cooke Aquaculture Inc. (Blacks Harbour, NB, Canada) were initially held in the Dr Joe Brown Aquatic Research Building (Memorial University of Newfoundland and Labrador, MUN) in 3 m^3^ tanks for several months at 8–10°C. Thereafter, they were transferred to 0.8 m^3^ tanks in the Annex Tank Room at the Ocean Science Centre of MUN. The tanks were initially supplied with flow-through seawater (∼32 ppt salinity) with temperature and oxygen levels maintained at 10°C and >95% of air saturation, respectively, and with an 8 h light:16 h dark photoperiod. There were three tanks containing 35 fish (∼675 g) and 2 weeks post-transfer, temperatures were decreased at 1°C per week to their respective acclimation temperature (8, 4 or 1°C). All fish were then held at these temperatures for ≥3 weeks (total acclimation ranged between 3 and 12 weeks). A custom-built glycol chiller made by Technical Services at MUN was used to supply the tanks with seawater <5°C. Temperature was not lowered below 1°C as it was difficult to maintain these temperatures in the current system. All fish were fed a commercial salmon diet (5 mm, EWOS, Surrey, BC, Canada) by hand at ∼1% body mass (or until satiation for fish kept at temperatures <4°C as they exhibited reduced feeding behaviour and appetite) 3 times a week. Fish were fasted for 24–48 h prior to surgery.

### Surgical procedures and recovery

Fish were netted from their tank and anaesthetized in oxygenated seawater containing tricaine methanesulfonate (TMS, 0.2 g l^−1^; Syndel Laboratories Ltd, Qualicum Beach, BC, Canada) until ventilatory movements ceased. Mass (g) and fork length (cm) were measured, and then the fish were placed supine on a wetted foam pad upon a surgical table where their gills were continuously irrigated with oxygenated seawater containing a maintenance dose of TMS (0.1 g l^−1^) at temperatures similar to those of acclimation (8, 4 or 1°C). Each fish was fitted with a dorsal aortic cannula (PE 50, Clay-Adams, Becton Dickensen and Co., Sparks, MD, USA) as in Smith and Bell (1964) and Gamperl et al. (1994) to allow for blood collection at various sampling points during the experiment (see below). Salmon were then placed on their right side and a 1.3 mm diameter Doppler^®^ flow probe (Model ES Cuff-type Transducer, 20 MHz, Iowa Doppler Products, Iowa City, IA, USA) was fitted around the ventral aorta as described in Gamperl et al. (1994). Lastly, the flow probe lead was connected to a directional pulsed Doppler^®^ flow meter (Model 545C-4, Bioengineering, University of Iowa, Ames, IA, USA) to ensure that the signal was of high quality, and the probe lead was secured to the fish at four locations using 2-0 silk suture: to the ceratobranchial element lining the posterior margin of the fourth buccal–opercular opening which is absent of gill filaments; just posterior to the pectoral fin; just below the lateral line; and just anterior to the dorsal fin.

After surgery was completed, each fish (*n*=7–9 per group) was transferred to a 81 l Blazka-type swim-tunnel respirometer (University of Waterloo, Biotelemetry Institute, Waterloo, ON, USA) with an internal diameter of 25 cm and a 90 cm long working section, that was filled with water of the appropriate temperature (8, 4 or 1°C). The front of the respirometer was fitted with a plastic grid, which allowed for uniform water flow in the swimming section of the respirometer (Taylor and McPhail, 1985), and the rear of the tunnel was fitted with a stainless steel grid that was connected to an external electrical circuit. This stainless steel grid could be electrified with a small current (∼0.2 A, <5 V); however, electrical stimulation to encourage swimming was only used during the experiment just prior to exhaustion as it interfered with the Doppler^®^ flow probe signal, particularly in fish tested at 1°C. The front of the tunnel was covered with black plastic to provide the fish with a dark refuge and to minimize stress from external stimuli (i.e. investigator presence). Seawater was supplied to the swim tunnel from a temperature-controlled 270 l water reservoir (MUN, Technical Services), and the O_2_ content of the water was maintained at >95% air saturation by bubbling the reservoir with air.

A ∼2 min ‘training session’ was performed >8 h post-surgery, during which the water velocity was gradually increased to ∼1 body length s^−1^ (BL s^−1^) to induce swimming and brought back down to 0.25 BL s^−1^ for recovery overnight. Finally, the temperature in the system was lowered overnight from 8°C to 1°C at ∼1°C h^−1^ for the acute exposure group, and maintained at this temperature until the swim trial began.

### Critical swim speed test

**Fig. 1.**
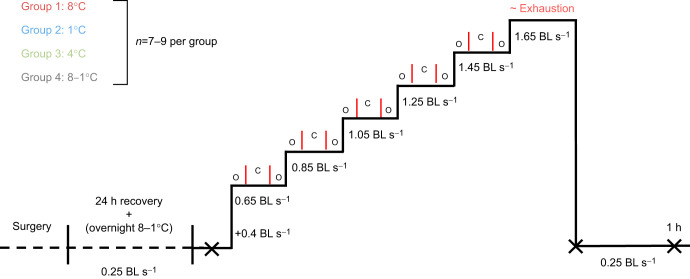
**Schematic diagram of the experimental design used to determine the critical swim speed (*U*_crit_) of Atlantic salmon exposed to cold temperatures and its effect on their cardiorespiratory and stress physiology.** Three groups of fish were acclimated to 8, 4 or 1°C and tested at their respective acclimation temperature, and a fourth group was acclimated to 8°C and tested at 1°C (acute cold exposure). The total time at each swim speed was 15 min and was composed of: a 5 min open (O) period; a 3–10 min closed (C) period to allow the drop in the partial pressure of oxygen (*P*_O_2__) to be recorded; and a final open (O) period to allow for percentage saturation to return to >85%. Cardiorespiratory parameters [heart rate (*f*_H_), stroke volume (*V*_S_), cardiac output (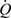) and oxygen consumption (*Ṁ*_O_2__)] were recorded at rest, at each increase in swim speed, immediately after exhaustion, and 1 h post-exhaustion. Blood samples for various haematological parameters were taken as indicated by ‘X’ (i.e. at rest, immediately after exhaustion and 1 h post-exhaustion).

Resting, active and post-exhaustion cardiorespiratory and metabolic parameters were recorded for all individual fish during a critical swim speed (*U*_crit_) test (Brett, 1964). After resting cardiac function and *Ṁ*_O_2_ _were measured at a baseline speed of 0.25 BL s^−1^, the swim speed was initially increased by 0.4 BL s^−1^ to induce constant swimming, followed by increments of 0.2 BL s^−1^ every 15 min until the fish were exhausted. Exhaustion was determined as the inability of the fish to move away from/off the electric grid after 2–3 successive mild (5 V) shocks and the swim speed was immediately reduced back to the baseline level (0.25 BL s^−1^) for 1 h (Fig. 1). The fish's critical swim speed was calculated as:
(1)

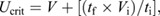
where *V* is the velocity at which the fish swam for the entire time increment; *V*_i_ is the velocity increment (0.2 BL s^−1^); *t*_f_ is the time elapsed from the last change in current velocity to fatigue; and *t*_i_ is the time increment (i.e. the time between increases in velocity or 15 min). Then, *U*_crit_ was corrected for the solid blocking effect of the fish (Bell and Terhune, 1970; Kline et al., 2015):
(2)

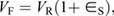
where *V*_F_ is the water velocity at the position of the fish's maximum girth; *V*_R_ is the water velocity at the rear of the flume; and ∈_S_ is the error due to solid blocking, which was calculated as:
(3)

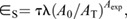
where τ is a dimensionless factor for tunnel cross-sectional shape (0.8); λ is a factor (coefficient) for the shape of the fish, set at 0.5 (i.e. the value for a fish with streamlined shape); *Α*_0_ is the cross-sectional area of the fish, calculated as 0.25*G*^2^π^−1^, where *G* is the maximum girth to the closest mm; *A*_T_ is the cross-sectional area of the swimming chamber, calculated as π*r*^2^, where the radius (*r*) was 100 mm; and *A*_exp_ is the fractional area exponent (1.5) (Kline et al., 2015).

### Metabolism and cardiorespiratory function

Oxygen consumption (in mg O_2_ kg^−1^ h^−1^) was measured using intermittent closed respirometry (Sandblom et al., 2014), and using methods consistent with recommendations for aquatic respirometry as detailed in Rodgers et al. (2016), Svendsen et al. (2016) and Killen et al. (2021). Oxygen level (*P*_O_2__) in the swim tunnel was continuously measured using a fibre-optic sensor (dipping probe) connected to a PreSens O_2_ meter (PreSens Precision Sensing GmBH, Resenberg Germany). The fish's *Ṁ*_O_2__* *was measured by manually stopping the flow of water into the swim tunnel, and by using LabChart v.8.1.5 (ADInstruments, Dunedin, New Zealand) to calculate the slope of the decrease in *P*_O_2__ after a 2 min wait period (i.e. when the tunnel was closed, which varied between 5 and 10 min depending on temperature to ensure an acceptable *r*^2^). An *r*^2^ value >0.7 for the decline in water O_2_ level with time was used because of the large signal to noise ratio exhibited by fish at colder temperatures (i.e. at 4 and 1°C) when at rest or during measurements of resting metabolic rate (RMR; see below), to ensure that *Ṁ*_O_2_ _was not overestimated (Chabot et al., 2020). Standard metabolic rate (SMR) was calculated by plotting the relationship between the log of metabolism [i.e. from RMR to maximum metabolic rate (MMR)] and swim speed (BL s^−1^), and extrapolating back to a swim speed of 0 BL s^−1^. Aerobic scope (AS) and ‘realistic’ aerobic scope (AS_R_) were then calculated as the difference between MMR and SMR, and MMR and RMR, respectively. Background measurements of *Ṁ*_O_2_ _were made when the tunnel did not contain a fish at the end of the experiments, and these were negligible (<1%), indicating that no substantial microbial respiration was occurring (Rodgers et al., 2016; Svendsen et al., 2016).

*f*_H_ and cardiac output were recorded by connecting the flow probe leads to a pulsed Doppler^®^ flow meter and signals from the Doppler^®^ flow meter were amplified and filtered using a data acquisition system (MP100A-CE; BIOPAC Systems, Inc., Santa Barbara, CA, USA) and a universal interface module (UIM100C, BIOPAC Systems, Inc.) connected to a laptop computer running AcqKnowledge^®^ software (v.3.8.2; BIOPAC Systems, Inc.). *f*_H_ (beats min^−1^) was determined by measuring the number of systolic peaks during two 30 s intervals while the system was closed for respirometry and values for 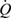 were recorded in volts (V). After the *U*_crit_ test was completed, all fish were euthanized using a lethal dose of MS-222 (0.4 g l^−1^), and an *in situ* post-mortem calibration of each flow probe was performed at physiologically relevant pressures using a peristaltic pump (MasterFlex EasyLoad^®^, Quebec, QC, Canada) (Gamperl et al., 1994) and a ‘blood mimicking’ solution (0.99% glycerol, 2.4% Triton X-100, 35% Orgasol in 200 ml of distilled water; M. Axelsson, University of Gothenburg, personal communication). Briefly, the sinus venosus and atrium were removed, the ventricle was bisected laterally, and a steel cannula attached to the peristaltic pump tubing was tied into the ventricular lumen. This allowed 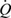 in volts to be converted to ml min^−1^ kg^−1^, *V*_S_ to be calculated as 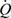/*f*_H_ (in units of ml kg^−1^) and oxygen extraction to be calculated as *Ṁ*_O_2__/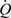 (in mg O_2_ ml^−1^ blood). In addition, the fractional change in a rate over a 10°C range (i.e. *Q*_10_ value) was calculated as an index of the effect of chronic (acclimation to 8 versus 1°C) and acute (8 to 1°C) temperature (*T*) changes on *Ṁ*_O_2__, *f*_H_, 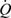, *V*_S_ and *Ṁ*_O_2__/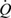 (*R*) using the following equation:
(4)

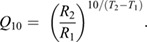
After the calibration was completed, the two halves of the ventricle were weighed, and the mass, fork length, condition factor and sex were recorded for all fish used in the experiment. Finally, relative ventricular mass (RVM) was calculated as:
(5)

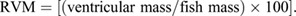
The morphometric data are shown in Table 1, and all data were examined statistically for any significant sex effects (see Tables S1 and S2).

**Table 1.**
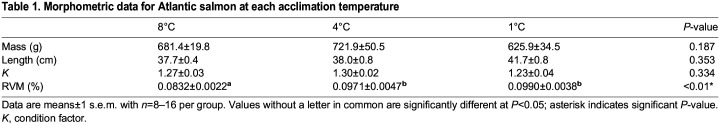
Morphometric data for Atlantic salmon at each acclimation temperature

### Haematological parameters

Blood samples (0.5 ml) were withdrawn from the dorsal aortic cannula and immediately replaced with 0.5 ml of saline at three time points: (1) 24 h post-surgery (i.e. prior to an increase in swim speed); (2) immediately after the fish were exhausted; and (3) 1 h after exhaustion. Blood samples were first drawn into microhaematocrit tubes and centrifuged at 10,000 ***g*** for 2 min to determine haematocrit (Hct; % of red blood cells). A 50 μl aliquot of whole blood was then collected for the measurement of blood haemoglobin (Hb) concentration using the cyanomethaemoglobin method (Drabkins reagent, D5941, Sigma-Aldrich, Oakville, ON, Canada) and the absorbance was read at 540 nm using a plate reader (SpectraMax M5e, Molecular Devices, San Jose, CA, USA). Hb concentrations were calculated from standard curves using bovine Hb (Sigma, H2500). Mean cellular Hb concentration (MCHC; mg ml^−1^) was calculated as ([Hb]/Hct)×100. The remaining blood sample was centrifuged for 1 min at 10,000 ***g*** in a mini-centrifuge (05-090-128, Fisher Scientific), and 25 μl aliquots of plasma were pipetted into Eppendorf^®^ tubes for the measurement of plasma cortisol and lactate levels. All samples were immediately frozen in liquid N_2_ and stored at −80°C. Cortisol was measured using an enzyme-linked immunosorbent assay (ELISA) kit (Neogen Life Sciences, 402710, Lexington, KY, USA) and a SpectraMax M5e microplate reader at 650 nm. Plasma lactate samples were first deproteinized with 6% (v/v) perchloric acid, then lactate was measured spectrophotometrically at 340 nm using the production of NADH/NADPH by lactate dehydrogenase (Sigma-Aldrich L2500), and lactate concentration was calculated with reference to standard curves (Sigma-Aldrich L6402).

### Statistical analyses

A Rosner's test [*EnvStats* package in R with α=0.05; https://CRAN.R-project.org/package=EnvStats (Millard, 2013)] and a Grubb's test (*outliers* package in R with α=0.05; http://CRAN.R-project.org/package=outlier) were used to detect outliers in all datasets prior to statistical analysis. These tests revealed that the lactate concentration for fish 7 in the 8°C-acclimated group was an outlier at the third sampling point (i.e. 1 h post-exercise) and it was removed from the dataset. Then all data were tested for assumptions of normality and homogeneity of variance using Shapiro–Wilks and Levene's tests, respectively (Fox and Weisberg, 2019). A general linear model (lm function) and ANOVAs (anova function) were used in the *stats* package of R to assess the effect of acclimation temperature on morphometric variables [mean mass (g), fork length (cm), condition factor (*K*) and RVM]. Similar models were used to assess how pre-test temperature conditions affected all resting and maximum cardiorespiratory, metabolic and *U*_crit_ values. If there was a significant effect, a Tukey's HSD *post hoc* test (*stats* package in R) was used to examine where the differences occurred. A general linear mixed model [lmer function in the *lme4* package https://CRAN.R-project.org/package=lme4 (Bates et al., 2015) and the *lmerTest* package https://CRAN.R-project.org/package=lmerTest (Kuznetsova et al., 2017) in R] was used to analyse all haematological parameters, including using ‘fish’ as a random factor, and ‘group’, ‘sampling point’ and their interaction as fixed effects. Main effects were analysed using ANOVA (anova function) with type III sums of squares, and if the model indicated a significant fixed effect, a Tukey's HSD *post hoc­* test identified statistical differences. All models for morphometric, cardiorespiratory and metabolic responses were separated by sex (male versus female) and whether there were significant effects of sex was examined. No significant sex effects were found.

All statistical analyses were performed using RStudio v.1.3.1093 (https://posit.co/products/open-source/rstudio/) with R v.4.1.0 (http://www.R-project.org/), and all data in the text, figures and tables are means±1 s.e.m. The threshold used for determining statistical significance was *P*<0.05; however, we also report where 0.08>*P*>0.05.

## RESULTS

### Morphometric parameters and swimming capacity

There were no differences in the mean mass, length or condition factor between acclimation groups (Table 1). However, fish acclimated to both 4 and 1°C had a significantly greater RVM as compared with those acclimated to 8°C (by 17% and 19%, respectively). No significant differences in the morphometric data were evident between male and female salmon at any acclimation temperature (Tables S1 and S2).

The *U*_crit_ of salmon decreased as acclimation temperature was reduced (i.e. from 2.08 BL s^−1^ at 8°C, to 1.69 BL s^−1^ at 4°C, and to 1.27 BL s^−1^ at 1°C), in accordance with the data for the fish's metabolic capacity (see below). However, the swimming capacity of fish acclimated to 1°C was unexpectedly, and significantly, lower than that of those acutely cooled to this temperature (1.44 BL s^−1^) (Table 2).

**Table 2.**
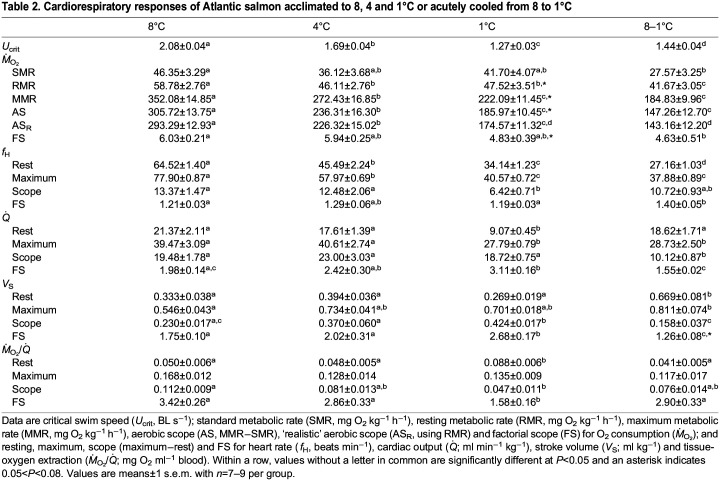
Cardiorespiratory responses of Atlantic salmon acclimated to 8, 4 and 1°C or acutely cooled from 8 to 1°C

### Metabolism

There were no significant differences in SMR between acclimation groups. However, when fish were acutely cooled from 8 to 1°C they had a 40% lower SMR than that of fish acclimated to 8°C (*P*<0.01), and this parameter was 35% lower than that measured in 1°C-acclimated fish (although this was not significant). RMR was also significantly lower (by ∼25%) in fish chronically and acutely exposed to cold temperatures (≤4°C) than in those acclimated to 8°C (*P*<0.01, Table 2). MMR was reduced significantly as acclimation/test temperature was decreased (i.e. the MMR for 4 and 1°C-acclimated salmon and those acutely exposed to 1°C was only 22%, 35% and 50% of that for salmon swum at 8°C). While there was no significant difference in MMR or AS_R_ between the two groups tested at 1°C (*P*=0.37), acutely cooled fish had *Q*_10_ values for RMR, SMR and MMR that were consistently higher than those of fish acclimated to this temperature (Table 3).

**Table 3.**
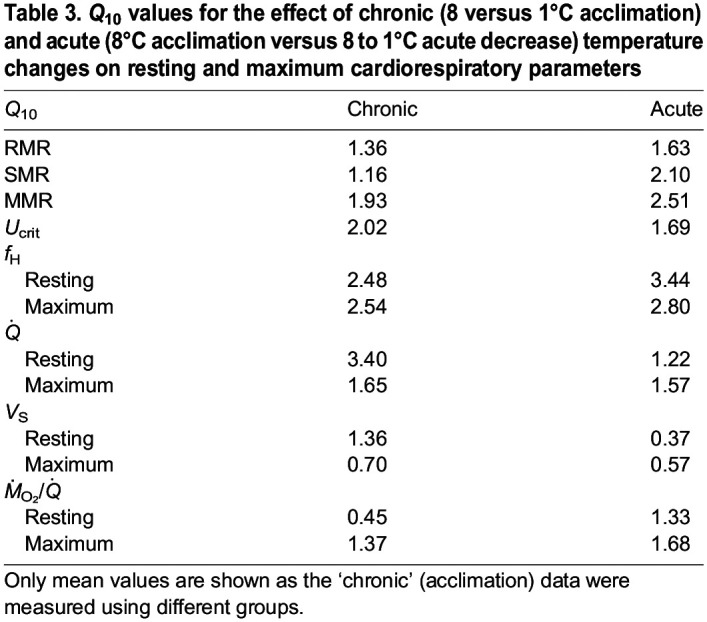
*Q*_10_ values for the effect of chronic (8 versus 1°C acclimation) and acute (8°C acclimation versus 8 to 1°C acute decrease) temperature changes on resting and maximum cardiorespiratory parameters

### Cardiac function

**Fig. 2.**
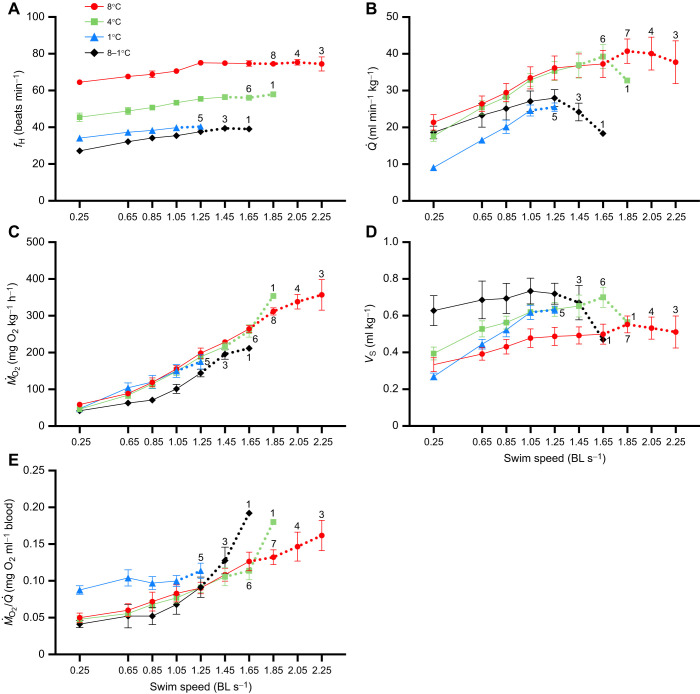
**The cardiorespiratory responses of Atlantic salmon acclimated to 8, 4 and 1°C or acutely cooled from 8 to 1°C and then subjected to a *U*_crit_ test.** (A) *f*_H_, (B) 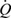, (C) *Ṁ*_O_2__, (D) *V*_S_ and (E) oxygen extraction (*Ṁ*_O_2__/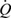) at each step during the *U*_crit_ test, starting at an initial (resting) speed of 0.25 BL s^−1^. Dotted lines indicated the speed when the number of fish (*n*) was reduced, and numbers above particular points indicate the remaining number of fish that made it to that swim speed within a specific group. Values are means±1 s.e.m. with *n*=7–9 per group, unless otherwise indicated.

Resting *f*_H_ decreased with acclimation temperature and was significantly lower in fish acutely exposed versus acclimated to 1°C (64.5±1.4 versus 45.5±2.2 versus 34.1±1.2 versus 27.2±1.0 beats min^−1^, *P*<0.0001, in 8, 4 and 1°C-acclimated and 8–1°C salmon, respectively) (see Table 2 and Fig. S1). Maximum *f*_H_ (*f*_H,max_) during exercise was also significantly lower in colder fish (8 versus 4 versus 1°C); however, there was no significant difference in *f*_H,max_ between the fish acclimated versus acutely exposed to 1°C (40.6±0.7 versus 37.9±0.9 beats min^−1^, respectively). Overall, fish exposed to temperatures ≤8°C had a very limited scope to increase *f*_H_ when challenged with the additional energetic demands of exercise (average scope ranged from ∼6.5 to 13.5 beats min^−1^; Fig. 2A). However, it appears that when temperature is rapidly decreased (i.e. at 1°C h^−1^) from 8 to 1°C, salmon have a significantly larger factorial scope to increase *f*_H_ with exercise as compared with those acclimated to ≤8°C (Table 2).

Resting 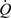 was significantly lower in 1°C-acclimated salmon than in 4 and 8°C-acclimated and acutely cooled fish (*P*<0.0001) (see Fig. 2B and Table 2). Further, maximum 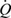 (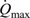) for 1°C-acclimated and acutely exposed fish were similar (*P*=0.991) and only ∼70% of the 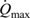 for 8 and 4°C- acclimated fish. Interestingly, fish acclimated to 1°C had the greatest capacity to increase 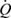 during exercise (factorial scope of 3.11), double that measured in acutely cooled fish (1.55). This was because: these fish had a significantly higher factorial scope for *V*_S_ (*P*<0.0001) than measured in all the other groups; and resting *V*_S_ for fish acutely exposed to 1°C was comparable to the maximum *V*_S_ for 1°C-acclimated fish (0.70±0.08 versus 0.70±0.02 ml kg^−1^) and, therefore, acutely cooled fish had a limited scope to increase their *V*_S_ during exhaustive exercise (Fig. 2D).

In contrast to the effects of acclimation to 1°C on *V*_S_ and 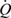, chronic exposure to this temperature resulted in significantly higher values for resting oxygen extraction [*Ṁ*_O_2__/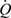 (0.088±0.0058 mg O_2_ ml^−1^ blood, *P*< 0.0001] in this group compared with those acclimated to 8 and 4°C (0.050±0.0062 and 0.048±0.0047 mg O_2_ ml^−1^ blood) and to fish acutely exposed to 1°C (0.041±0.0050 mg O_2_ ml^−1^ blood). However, the 1°C-acclimated fish had no scope to increase oxygen extraction and, thus, the maximum value in this group was statistically similar to that of the other groups (mean value of 0.137±0.011 mg O_2_ ml^−1^ blood) at peak exercise; whereas fish acutely decreased from 8 to 1°C had a significantly greater scope (∼2-fold) to increase *Ṁ*_O_2__/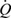 than those acclimated to 1°C when swum to exhaustion (Table 2).

### Haematological parameters

**Fig. 3.**
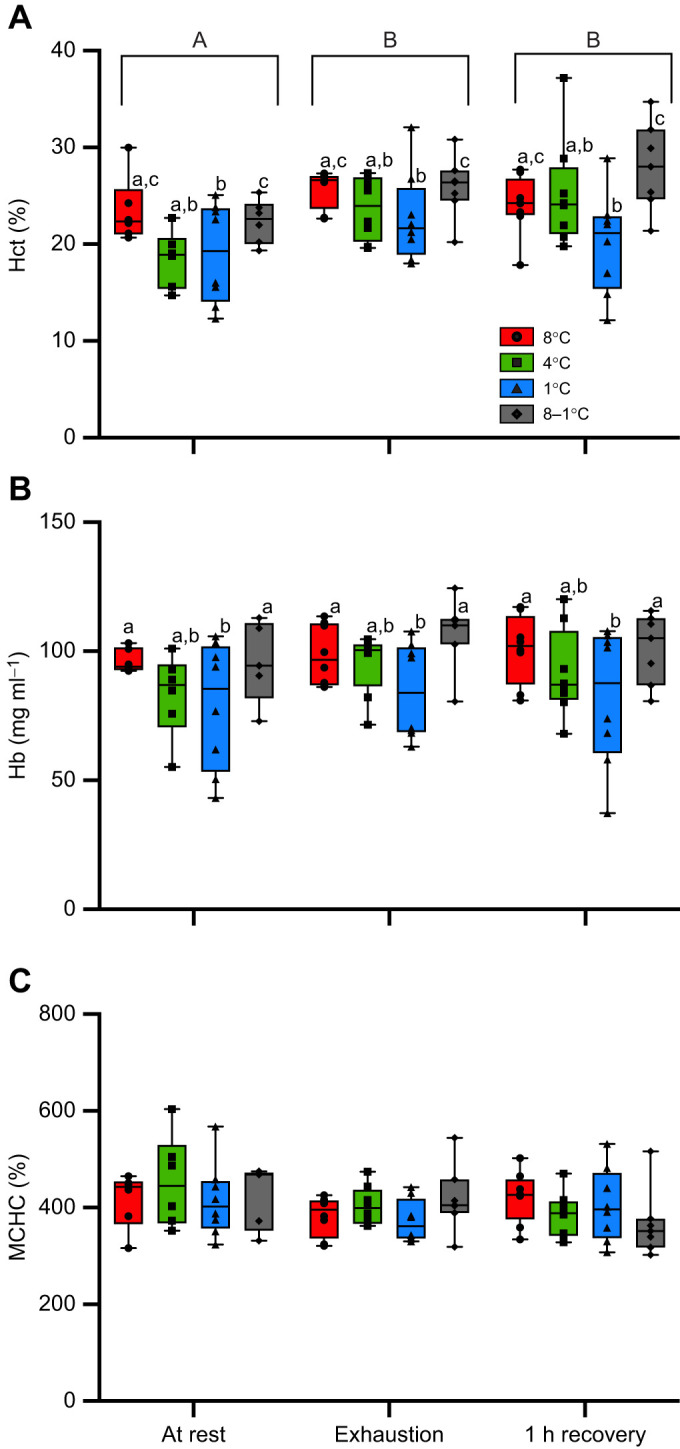
**The haematological responses of Atlantic salmon acclimated to 8, 4 and 1°C or acutely cooled from 8 to 1°C and then subjected to a *U*_crit_ test.** Data were obtained prior to (at rest), at the end of (exhaustion) and 1 h after (1 h recovery) the *U*_crit_ test. Box plots show median (horizontal line), upper and lower quartiles (box) and the 1.5× interquartile range (whiskers). (A) Haematocrit (Hct), (B) haemoglobin concentration ([Hb]) and (C) mean cellular Hb concentration (MCHC). Values without a letter in common are significantly different (*P*<0.05) between groups at a particular sampling point (lowercase letters) and between sampling points within a group (uppercase letters). Values are means±1 s.e.m. with *n*=6–8 per group.

There was a significant effect of both group and sampling point on Hct (Fig. 3A). On average, Hct was lower in the 4 and 1°C-acclimated salmon (by ∼3.5%) as compared with those acclimated to 8°C and acutely cooled from 8 to 1°C. All groups had elevated Hct immediately after exhaustion and 1 h post-exercise as compared with resting values; this increase ranged from 0.61% to 6.77%. There were no significant differences in [Hb] between the various sampling points. However, on average, fish acclimated to 1°C had a significantly lower [Hb] than both the 8°C-acclimated and acutely cooled fish at each sampling point (by ∼18%; Fig. 3B). No significant differences in MCHC were observed throughout this experiment, with values ranging from approximately 325 to 475 ng ml^−1^ (Fig. 3C).

**Fig. 4.**
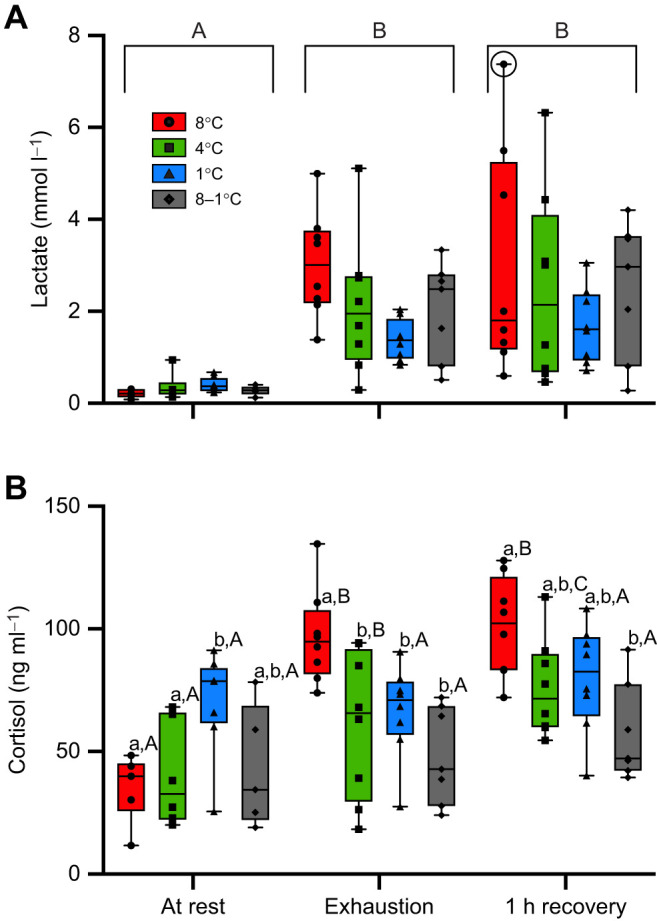
**Changes in plasma lactate and cortisol levels in Atlantic salmon acclimated to 8, 4 and 1°C or acutely cooled from 8 to 1°C and then subjected to a *U*_crit_ test.** Data were obtained prior to (at rest), at the end of (exhaustion) and 1 h after (1 h recovery) the *U*_crit_ test. (A) Plasma lactate. (B) Plasma cortisol. Values without a letter in common are significantly different (*P*<0.05) between groups at a particular sampling point (lowercase letters) and between sampling points within a group (uppercase letters). The data point in A that is circled represents an outlier and was removed from statistical analysis. Values are means±1 s.e.m. with *n*=5–8 per group.

Plasma lactate levels increased significantly in all fish during exercise and were still elevated 1 h after the fish reached exhaustion (*P*< 0.0001), rising on average from 0.32±0.04 mmol l^−1^ at rest to 2.14±0.34 and 2.43±0.27 mmol l^−1^, respectively (Fig. 4A). Although there were no significant differences in lactate levels between the groups, plasma lactate levels increased by ∼4-fold in fish acclimated to 1°C between rest and 1 h post-exhaustion, whereas the magnitude of the increase in fish acclimated to 8 and 4°C and acutely cooled to 1°C was ∼14-, 7- and 9-fold, respectively. Acclimation and sampling point had an interactive effect (*P*< 0.0001) on plasma cortisol concentration (Fig. 4B). Resting plasma cortisol concentration (i.e. 24 h post-surgery and when confined within a swim tunnel) was significantly higher in fish acclimated to 1°C than in both 8 and 4°C-acclimated fish (70.6±7.4 versus 34.9±6.0 and 40.3±8.7 ng ml^−1^, respectively, *P*< 0.05). However, these values were all statistically similar to those in fish acutely cooled to 1°C (43.2±11.1 ng ml^−1^). Neither group of salmon tested at 1°C had elevated plasma cortisol concentrations just after exhaustion or following a 1 h post-exercise recovery, with levels similar to those at rest. However, 4 and 8°C-acclimated fish (which had lower resting levels) experienced 2- and 3-fold increases in plasma cortisol concentration at 1 h post-exhaustion (i.e. to 100.9±7.2 and 76.0±7.0 ng ml^−1^, respectively).

## DISCUSSION

Predictably, low temperatures (1, 4 and 8–1°C) decreased the salmon's cardiorespiratory and swimming capacity. However, this study also reports several novel findings that have important implications for our understanding of fish temperature-dependent cardiorespiratory physiology. First, increases in *f*_H_ with swimming were very limited (20–40%) in our salmon, and these data support the recent findings of Gamperl et al. (2022) that suggest myocardial contraction and twitch kinetics greatly constrain maximal *f*_H_ in fishes at cold temperatures. Second, while the higher resting *f*_H_ in 1°C-acclimated salmon as compared with that of salmon acutely exposed to this temperature was not unexpected (Aho and Vornanen, 2001; Haverinen and Vornanen, 2007; but also see below), the fact that these two groups used completely different mechanisms to increase blood oxygen delivery/*Ṁ*_O_2__ when exercised (*V*_S_ and 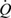 versus tissue oxygen extraction, respectively) is particularly notable. Specifically, these data suggest that while salmon have considerable plasticity with regard to meeting increased oxygen demands at cold temperatures, the mechanisms evoked differ based on how long they have been in the cold (i.e. they appear to increase either 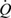 or 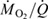 when swimming, but not both). Third, given that measurements of maximum cardiac function and metabolic capacity (i.e. 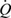, MMR and AS_R_) were not different (or marginally higher) in salmon acclimated to 1°C as compared with those of fish acutely exposed to this temperature (Table 2), it is clear that factors independent of systemic (total) blood oxygen transport limit swimming capacity in salmon when chronically exposed to temperatures approaching their lower limit.

### Heart size, blood oxygen carrying capacity and stress at cold temperatures

#### RVM

In this study, salmon acclimated to 4 and 1°C had much larger (by 17–20%; Table 1) ventricles as compared with those of 8°C-acclimated fish. An increase in relative ventricular mass has been observed in many fish populations (including non-polar temperate species) that exploit cold water habitats during their life history. However, this is not a universal response for all fish species and may differ between sexes and families (Aho and Vornanen, 2001; Axelsson, 2005; Driedzic et al., 1996; Farrell et al., 1988; Gamperl and Farrell, 2004; Graham and Farrell, 1989; Kent et al., 1988; Klaiman et al., 2011). For example, while cardiac/ventricular remodelling (i.e. hypertrophy) has been well documented in many salmonids to support cardiac function and ensure the adequate delivery of oxygen to tissues at cold temperatures, Graham and Farrell (1992) showed that rainbow trout raised in confined domesticated conditions (i.e. where temperature was held constant at 8–11°C all year) had smaller values for RVM as compared with wild fish where temperatures ranged seasonally from 4 to 15°C. Further, a previous study by Porter et al. (2022), which used a population of Atlantic salmon raised in land-based systems and held at temperatures from 6 to 15°C, did not report an increase in RVM with cold acclimation, while salmon acclimated to the same temperatures in the present study (derived from a population of salmon selected for performance in sea-cages where environmental temperatures fluctuate between 0 and 20°C) had a much greater RVM. These differing responses between salmon raised in land-based systems versus in conditions where environmental parameters fluctuate considerably, ultimately support the findings of Graham and Farrell (1992) and Morgan et al. (2022), and suggest that the phenotypic ‘plasticity’ of salmonids at cold temperatures may, at least in part, be related to their long-term thermal history.

#### Hct and Hb

Changes in haematology are common at cold temperatures (0–5°C), and have been well described for many polar and Antarctic species (Farrell and Steffensen, 2005; Holeton, 1970; Ruud, 1954). An increase in blood viscosity is a common challenge for fish exposed to chronically cold temperatures, and this is typically counteracted by a reduction in, or in the case of some Antarctic fishes the absence of, Hct and [Hb] (Axelsson, 2005; Egginton, 1996), which in turn lowers blood oxygen carrying capacity. However, the small decrease in Hct in salmon acclimated to 4 and 1°C (∼3.5%) suggests that this response may not have a significant impact at cold temperatures given their low RMR and MMR (e.g. Table 2).

Sufficient oxygen delivery (Hct and [Hb]) to the tissues has been proposed as a key determinant of thermal tolerance, and blood oxygen carrying capacity is particularly important in this regard when fish are exposed to acute stress or other challenges (Anttila et al., 2013; Leeuwis et al., 2021; Muñoz et al., 2018; Pörtner and Knust, 2007; Pörtner, 2010; Wang and Overgaard, 2007). The release of red blood cells (RBCs) from the spleen is a common secondary stress response that enhances blood oxygen carrying capacity (Muñoz et al., 2018; Pearson and Stevens, 1991; Sandblom and Axelsson, 2007), and occurred in all groups of fish during exercise based on the elevated Hct (% RBCs); although swimming did not change [Hb] or MCHC. Nevertheless, it does not appear that time spent at 1°C impacts the salmon's capacity to release erythrocytes from the spleen at exhaustion (Fig. 3) or to increase blood oxygen carrying capacity. The differences in Hct and [Hb] between fish at rest and at exhaustion were of a similar magnitude.

#### Elevated cortisol levels

Resting values for plasma cortisol (i.e. prior to the *U*_crit_ test) were significantly higher in salmon acclimated to 1°C versus 4 or 8°C, and this is consistent with the findings of a previous study on salmon chronically and acutely exposed to 1°C (Porter et al., 2022). Collectively, these data reinforce the notion that temperatures approaching a fish's lower thermal limit are stressful and suggest that exposure to these temperatures has consequences for the fish. The impact of a stressor is difficult to define, and is typically indicative not only of the duration of stress (i.e. acute versus chronic) but also of the overall duration and severity of the consequences following exposure (Boonstra, 2013; Schreck and Tort, 2016). However, it is likely that this level of stress would affect their ability to survive additional environmental stressors (see reviews by Shreck, 2000 and Schreck and Tort, 2016) and make them more susceptible to opportunistic infections. Cortisol is immunosuppressive (Maule et al., 1987; Pickering and Pottinger, 1989; Yada and Tort, 2016) and cold temperatures alone are known to negatively impact immune function (Abram et al., 2017; Guo and Dixon, 2021). Interestingly, whereas 4 and 8°C-acclimated fish had elevated plasma cortisol levels at exhaustion and during recovery, neither group of 1°C-exposed fish had higher plasma cortisol levels at these sampling points (Fig. 4B). Clearly, future research should examine the effects of cold acclimation and exposure on the secondary and tertiary stress responses of salmonids, and the possible adaptive versus maladaptive (see below) consequences for the fish's ability to perform maximally or to return to homeostasis post-stress.

### Cardiac function in response to cold temperatures

#### Acclimation effects on *f*_H_ and its limited scope during exercise

It has been shown that salmonids can compensate for the physiological effects of prolonged exposure (i.e. acclimation) to colder seasonal temperatures by having a higher routine *f*_H_ than would be observed if the fish were acutely exposed to these same temperatures (Aho and Vornanen, 2001; Gamperl and Farrell, 2004; Haverinen and Vornanen, 2007). Consistent with these findings, the temperature coefficient (*Q*_10_) for resting *f*_H_ in the present study was lower in fish acclimated versus acutely exposed to 1°C (Table 3), and this suggests that: (1) at least partial compensation had occurred in salmon acclimated to extremely low temperatures (i.e. approaching their lower thermal limit); and (2) the ‘resetting’ of *f*_H_ *in vivo* (i.e. in a fish with intact neuroendocrine control systems) takes longer than 8 h. This latter finding is consistent with Ekström et al. (2016) and suggests that we must be careful interpreting data from reduced (i.e. *in vitro*) or pharmacologically manipulated preparations (e.g. Sutcliffe et al., 2020; Gilbert et al., 2022; Sandrelli and Gamperl, 2023) with regards to how *f*_H_ changes (including its temporal nature) in free-living fish when exposed to varying environmental challenges. This study did not examine the mechanisms responsible for the resetting of *f*_H_, but the increase in resting *f*_H_ following acclimation to 1°C could have been the result of: a shift in cardiomyocyte membrane ion channel function and/or density (Vornanen, 1998; Vornanen et al., 2002a,b); an increase in adrenoreceptor sensitivity to extrinsic factors (i.e. circulating hormones adrenaline and noradrenaline) (Gamperl and Farrell, 2004); and/or a change in the cholinergic and adrenergic control of cardiac function (Ekström et al., 2016, 2021; Porter et al., 2022).

Interestingly, all fish regardless of acclimation or test temperature had a limited scope to increase *f*_H_ with exercise, and there was no difference in the *f*_H,max_ achieved between the groups tested at 1°C (Table 2). These results suggest that the ‘resetting’ of *f*_H_ to a higher level with cold acclimation is not accompanied by an enhancement in the maximum achievable *f*_H_. Instead, it appears that the concomitant lower resting *V*_S_ (but interestingly, not the larger RVM given that maximum *V*_S_ was not different; Table 2) provides salmon with a large scope for *V*_S_ which can be used to meet increased demands for cardiac pumping and oxygen delivery to the tissues. For example, 1°C-acclimated fish had a scope for *V*_S_ and 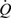 that was 2-fold that measured in those acutely exposed to this temperature and was considerably higher than that measured in fish acclimated to and tested at 8 and 4°C (Table 2). Clearly, cold exposure regardless of duration reduced the *f*_H,max_ reached when salmon were exercised to exhaustion, and we now have some insights into the underlying mechanism(s) mediating this effect. For example, Gamperl et al. (2022) provide convincing data which suggests that myocardial contraction and twitch kinetics greatly constrain *f*_H,max_ in rainbow trout at cool temperatures.

#### Phenotypic plasticity in salmon at temperatures approaching their lower thermal limit

The effect of cold acclimation on RVM and resting *f*_H_, combined with the different responses of *V*_S_ and tissue oxygen extraction during exercise between Atlantic salmon acutely versus chronically exposed to 1°C (Fig. 2 and Tables 2 and 3), point to the considerable cardiorespiratory phenotypic plasticity that Atlantic salmon have to deal with changes in temperature that approach their lower thermal limit. As previously discussed, all fish had a limited scope to increase *f*_H_ during exercise at ≤8°C. However, as acclimation temperature decreased (from 8 to 4 to 1°C), their factorial scope for *V*_S_ and 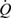 increased (Table 2) and, thus, 1°C-acclimated fish did not need to elevate *Ṁ*_O_2__/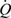 to meet the metabolic demands of exercise. On the contrary, fish acutely exposed to a drop in temperature to 1°C did not, or could not, increase *V*_S_ as this parameter was already elevated at rest, and when swum at this cold temperature *Ṁ*_O_2__/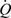 increased by 2.9-fold as compared with 1.6-fold in cold-acclimated fish (Table 2). Surprisingly, however, one might have anticipated that the cold (1°C)-acclimated salmon would have had a greater maximum *V*_S_ given their larger heart, and this value if anything was less than that observed in salmon acutely exposed to 1°C.

Interestingly, hypoxia-acclimated Atlantic salmon swimming at moderate speeds were able to increase 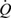 and *V*_S_ to the same extent as normoxia-acclimated fish, despite their lower RVM (Harter et al., 2019) and, thus, such studies raise the possibility that the increase in ventricle size when exposed to challenging environments may not be that beneficial with respect to Atlantic salmon being able to achieve maximal levels of cardiac pumping. However, there are other explanations for why the increased RVM of 1°C-acclimated salmon did not translate into a greater maximum *V*_S_ and 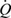. It is very likely that cortisol levels were chronically elevated in the 1°C-acclimated salmon (present study; Vadboncoeur et al., 2023), and it has been reported that cortisol-induced cardiac hypertrophy is maladaptive (pathophysiological) (Johansen et al., 2017). These cortisol-induced effects could have offset any potential benefits of hypertrophic growth at low temperatures on *V*_S_, stroke work and cardiac power output (Graham and Farrell, 1989). Alternatively, it could be that acclimation to the cold results in changes in central venous pressure (CVP) and/or its regulation, and that this limited maximum *V*_S_ in the 1°C-acclimated fish. Brijs et al. (2017) suggest that increases in CVP are critical with regard to achieving increases in end-diastolic volume, and *V*_S_, in seawater-acclimated rainbow trout. Unfortunately, there are no measurements of CVP in salmonids or other temperate fish species at temperatures below 10°C.

Acclimation to hypoxia has been reported to increase the role played by plasma-accessible carbonic anhydrase (paCA) in tissue oxygen delivery in swimming Atlantic salmon at 12°C (i.e. the role played by paCA in tissue O_2_ delivery shows plasticity), and it has been suggested that maximal exercise performance in salmon may not be possible without paCA (Harter et al., 2019). However, it was not fish acclimated to 1°C that showed a large scope for *Ṁ*_O_2__/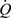; instead, it was the salmon acutely exposed to 1°C, which had little time to modulate paCA activity (e.g. through increases in protein levels or the isoforms expressed, especially given the cold temperatures), where large increases in *Ṁ*_O_2__/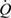 were observed. Further, it is unclear why the two groups only modulated *V*_S_ or *Ṁ*_O_2__/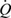, but not both. The latter is one of the main questions stemming from this work. Perhaps, like fish solely increasing *f*_H_ when exposed to increasing temperatures when they could achieve the same thermal tolerance and 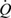 increase if they instead only increased *V*_S_ (and there theoretically would be benefits to this modulation of 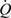; Keen and Gamperl, 2012), there are inescapable constraints on physiological regulation. For example, it is possible that fish at cold temperatures cannot increase *V*_S_ and *Ṁ*_O_2__/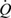 because an increase in O_2_ extraction would decrease the oxygen content of the venous blood perfusing the myocardium, and this ‘feedback’ prevents them from increasing together. Experiments where fish are exercised at cold temperatures while normoxic and hyperoxic would provide some insights here, as hyperoxia increases maximum 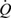 and *V*_S_ in temperate fish species (McArley et al., 2021, 2022).

### Effects of cold exposure on metabolism and swimming performance

#### Metabolic capacity at cold temperatures

Optimal temperatures for maximum aerobic scope have been well defined for fishes, with significant differences identified both between and within species when tested under various conditions (Casselman et al., 2012; Claireaux et al., 2000; Eliason and Anttila, 2017; Farrell, 2002; Hvas et al., 2017; Pörtner, 2001; Riseth et al., 2020; Taugbøl et al., 2019). However, most of the existing literature on salmonids has focused on determining thermal optima and upper tolerance limits, and their relationship to blood oxygen delivery and/or utilization (i.e. mitochondrial function) (Anttila et al., 2013, 2015; Beemelmanns et al., 2021; Gerber et al., 2020a,b), and this study is the first to quantify how acutely and chronically exposing this taxon to 1°C affects its basal metabolism (i.e. SMR and RMR) and metabolic capacity (i.e. MMR, AS and AS_R_). The much lower values for MMR, AS and AS_R_ in cold-acclimated fish were anticipated as we acclimated and tested salmon at temperatures far below their optimum for performance (∼13–20°C) (Brett, 1971; Handeland, et al., 2003, 2008; Hvas et al., 2017; Jonsson, et al., 2001; Keen and Farrell, 1994), and Hvas et al. (2017) showed that Atlantic salmon experience a large (∼30%) reduction in aerobic scope when acclimated to 3 versus 8°C. In our experiment, which tested salmon at even lower temperatures, we found a 40% reduction in aerobic scope (both AS and AS_R_) when salmon are acclimated to 1°C as compared with 8°C, and this suggests that aerobic scope becomes increasingly limited at such low temperatures.

The release of plasma lactate into the blood typically occurs at temperatures approaching the upper thermal limit of fishes as a result of a secondary stress response, or when aerobic metabolism is not sufficient to meet the fish's energetic needs (Clark et al., 2008; Eliason et al., 2013; Pörtner and Knust, 2007; Pörtner, 2010). Interestingly, studies examining the effects of cold shock on Atlantic salmon and common carp show that rapid decreases in temperature result in lower plasma lactate levels, and this suggests that fish at cold temperatures are supplied with sufficient levels of oxygen to meet their metabolic demands and that they do not experience mitochondrial dysfunction at these temperatures (Gerber et al., 2022; Porter et al., 2022; Tanck et al., 2000). In our study, resting plasma lactate concentration was not different between groups, and this supports the above conclusion. A switch from aerobic to anaerobic metabolism also occurs when fish reach approximately 70–80% of their *U*_crit_ (Burgetz et al., 1998), and as the fish swims faster there is a progressive increase in the recruitment of white (fast) muscle fibres, which rely on anaerobic energy sources (Brauner et al., 2000; Farrell, 2002; Hvas et al., 2017; Rodnick and Planas, 2016; Rome et al., 1992; Steinhausen et al., 2008). In the present study, there was no significant effect of acclimation or test temperature on the increase in plasma lactate levels between fish at rest and at exhaustion, with lactate increasing from 0.32 to 2.14 mmol l^−1^, on average, during the *U*_crit_ test. This increase in lactate is very similar to that for rainbow trout when swum at 9°C (0.31 to 1.97 mmol l^−1^; Brauner et al., 2000). The fact that there was no difference in plasma lactate levels between the groups is surprising given that the *U*_crit_ of 1°C fish was much lower than that of fish acclimated to 4 and 8°C. However, when fish are exposed to cold temperatures, the white (anaerobic) fibres are recruited at lower swimming speeds, possibly as a result of compromised red muscle function or oxygen delivery (Rome et al., 1985; Taylor et al., 1996), and, thus, white fibres probably make a similar overall contribution to the fish's maximum swimming capacity.

#### Swimming performance

Salmon in the current experiment exhibited ∼20%, 40% and 30% decreases in their *U*_crit_ when acclimated to 4 and 1°C and acutely exposed to 1°C, respectively, as compared with fish acclimated to 8°C. These reductions in *U*_crit_ in cold-acclimated fish are comparable to those for Atlantic salmon (30%) when acclimated to 10.5 versus 3°C (Riseth et al., 2020) and to the maximum sustainable swimming speed reported for rainbow trout acclimated to 11 versus 4°C (35%, Taylor et al., 1996), but slightly more than in Atlantic salmon acclimated to 8 versus 3°C (i.e. 12%; Hvas et al., 2017) or in wild brown trout (*Salmo trutta*) (i.e. 13%) when acclimated and tested at 5.5 versus 1.7°C (Taugbøl et al., 2019). However, it is difficult to discern why fish acclimated to 1°C had a lower *U*_crit_ than those acutely exposed to 1°C. Their MMR and AS_R_ were similar (if not higher) despite the slight decrease in Hct and blood [Hb]. Acclimation to cold temperatures has been reported to result in a number of morphological and physiological changes in many species that improve the functioning of the fish's red muscle – the muscle primarily powering sustained aerobic swimming. For example, it is typically reported that red muscle mass and fibre size (diameter) increase, the oxidative capacity of red muscle is greater, and cold acclimation results in changes in muscle mechanics that enable the muscle to produce more power (Egginton and Cordiner, 1997; Egginton and Sidell, 1989; Guderley, 2004; Johnston et al., 1990; Jones and Sidell, 1982; Rome and Swank, 2001). Further, and importantly, Gamperl and Syme (2021) reported that the red muscle of 6°C-acclimated Atlantic salmon was able to produce more power when tested at 2°C than that of 15°C-acclimated fish.

Nonetheless, there are a number of potential explanations for the lower swimming performance of 1°C-acclimated fish. First, cold acclimation is concomitant with an increase in muscle mass, but the capillary density of this tissue actually decreases (Egginton and Cordiner, 1997). Further, although Barron et al. (1987) did not report changes in red muscle blood flow with cold acclimation in rainbow trout, Taylor et al. (1996) reported a dramatic 93% (or 13.5-fold) decrease in tissue-specific blood flow (whereas cardiac output was only reduced ∼7-fold) when cold- versus warm-acclimated trout were swum to their maximum sustainable swimming speed. Thus, limited perfusion of the red muscle in 1°C-acclimated fish may have constrained its function and, thus, the fish's swimming capacity. Second, this is the first study to examine the swimming performance of a salmonid acclimated to a temperature close to its lower thermal limit (1°C), whereas the lower acclimation temperature used in previous studies was 3–4°C (e.g. Hvas et al., 2017; Taylor et al., 1996). While this temperature difference is small (2–3°C), Guderley and St Pierre (1999) indicate that there is a lack of compensation of oxidative metabolism when rainbow trout are seasonally acclimated to 0–2°C in winter. In addition, while Crockford and Johnston (1990) showed that the force development of fast muscle fibres increased when measured at 0°C in carp (*Cyprinus carpio*) acclimated to successively cooler temperatures (from 23 to 8°C), acclimating these fish to 2°C did not improve, or actually decreased, this parameter, i.e. there was no thermal compensation when these fish were acclimated to temperatures <8°C. Given that the rainbow trout (another salmonid) is considered to only have a moderate acclimatory response to cold temperatures as compared with cold-adapted species (Shuman and Coughlin, 2018), it is likely that the capacity of salmonids to perform at cold temperatures may be more limited than that of the carp. Finally, Cordiner and Egginton (1997) suggest that environmental factors other than temperature are likely to influence the nature of acclimatization in fish muscle. The carp acclimated to 2 and 5°C in Crockford and Johnston (1990) did not eat at these temperatures, and our salmon were not feeding much, if at all, at 1°C. This difference in energetic status could have negatively influenced the swimming performance of 1°C-acclimated fish. In addition, the salmon in this experiment were all held at the same photoperiod (8 h light:16 h dark), and it is not known how this might have affected the swimming performance of the different temperature-acclimated groups.

### Conclusions and perspectives

Overall, this study greatly extends our understanding of temperature-dependent effects on the cardiorespiratory function, metabolic capacity and swimming performance of Atlantic salmon. Our data reveal that: cold (1°C) acclimation results in an increase in RVM and resting *f*_H_, but not *f*_H,max_; Atlantic salmon have a limited capacity to increase *f*_H_ when swimming at cold temperatures; there are key differences in how 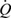 and *Ṁ*_O_2__/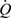 contribute to oxygen delivery to the tissues at rest and when swimming depending on whether fish are acutely (over hours) versus chronically (>3 weeks) exposed to temperatures approaching their lower thermal limit (0–1°C); and, finally, despite equivalent or higher values for MMR and AS_R_, 1°C-acclimated salmon had decreased swimming performance as compared with those acutely exposed to this temperature. Further, it is apparent that, based on plasma cortisol values measured in this and other studies, salmon are chronically stressed by prolonged exposure to 1°C.

This work provides some of the first *in vivo* data outlining the effects of very cold temperatures (i.e. approaching lethal lower temperature limits) on salmon cardiac function and swimming performance, and provides further evidence that climate variability (in this case ‘cold shocks’) has important implications for wild and aquaculture-reared salmonids. However, there is a lot more research that needs to be conducted before we can understand the significance, and mechanistic underpinnings, of the above findings. To start, it would be valuable to understand why cold-acclimated salmon did not achieve a greater maximum *V*_S_ during the *U*_crit_ test given that their hearts were considerably larger. Was it because the heart's function was compromised (possibly due to the pathophysiological effects of chronically elevated circulating cortisol levels), or that these fish had trouble increasing and/or regulating CVP and that this constrained *V*_S_? In this regard, measurements of CVP in fish acutely and chronically exposed to cold temperatures would be particularly valuable, as such measurements have only been made on salmonids acclimated to temperatures between 10 and 16°C. It would also be important to specifically examine the effects of cold temperatures on red muscle morphological and mechanical characteristics (e.g. using cycling muscle preparations; Gamperl and Syme, 2021) given the poor swimming performance of this species when acclimated to ∼0°C. Finally, in this study we calculated oxygen extraction indirectly as *Ṁ*_O_2__/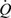. It is key that measurements of the salmon's venous and arterial blood oxygen content be made when they are exposed to acute and chronic changes in temperature to confirm the reported differences with regard to how 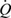 and *Ṁ*_O_2__/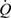 were utilized to increase oxygen delivery with exercise.

## Supplementary Material

10.1242/jexbio.245990_sup1 Supplementary information
