# Supplementary material for: Cardiorespiratory physiology and swimming capacity of Atlantic salmon (Salmo salar) at cold temperatures
Source: J Exp Biol. 2023 Sep 6;226(17):jeb245990. doi: 10.1242/jeb.245990 (PMC10499030; doi:10.1242/jeb.245990)
Supplement: Supplementary information [file jexbio-226-245990-s1.pdf]

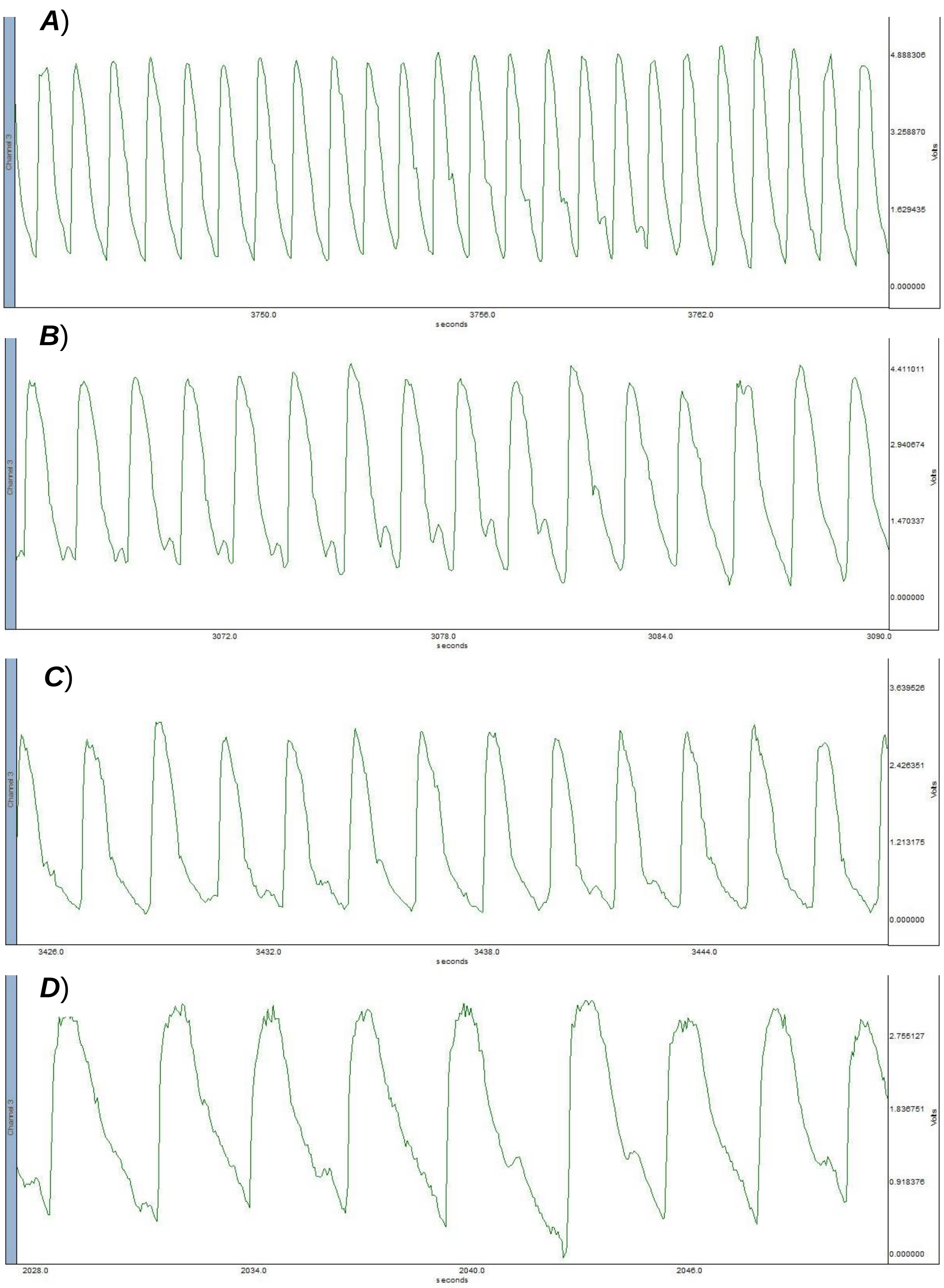


**Fig. S1. The resting heart rate ($f_H$; beats min$^{-1}$) of Atlantic salmon: (*A*) acclimated and tested at 8°C, (*B*) acclimated and tested at 4°C, (*C*) acclimated and tested at 1°C and (*D*) acclimated to 8 and tested at 1°C.** All tracings were recorded prior to a critical swim speed ($U_{crit}$) test.

**Table S1.** Morphometric data (± 1 s.e.m.) for all fish used in the experiment separated by acclimation temperature and sex. K = condition factor and RVM = relative ventricular mass. There was no significant effect of sex on any of the parameters. Dissimilar letters indicate significant ($p < 0.05$) differences between acclimation temperatures.

| | **8°C** | **4°C** | **1°C** |
|---|---|---|---|
| Weight (g) | | | |
| *Male* | 717.1 ± 22.4 | 745.8 ± 99.0 | 665.6 ± 37.7 |
| *Female* | 653.7 ± 21.01 | 689.0 ± 41.7 | 559.7 ± 53.6 |
| Length (cm) | | | |
| *Male* | 38.1 ± 0.5 | 38.3 ± 1.5 | 37.7 ± 0.8 |
| *Female* | 37.3 ± 0.5 | 37.6 ± 0.6 | 35.0 ± 1.3 |
| K | | | |
| *Male* | 1.29 ± 0.042 | 1.30 ± 0.036 | 1.24 ± 0.039 |
| *Female* | 1.26 ± 0.032 | 1.31 ± 0.026 | 1.21 ± 0.113 |
| RVM (%) | | | |
| *Male* | 0.0857 ± 0.0030[a] | 0.0926 ± 0.0056 [b] | 0.0968 ± 0.0058 [b] |
| *Female* | 0.0812 ± 0.0030 [a] | 0.102 ± 0.0075 [b] | 0.103 ± 0.0038 [b] |

Journal of Experimental Biology • Supplementary information

**Table S2.** Results of the two-way ANOVA that examined the effects of the fixed factors of acclimation temperature and sex on salmon morphometric parameters.

| **Morphometric** | **Main Effect** | **Df** | **F** | ***P*** |
|---|---|---|---|---|
| Weight | Acclim | 2 | 1.962 | 0.159 |
| | Sex | 1 | 3.984 | 0.0557 |
| | Acclim x Sex | 2 | NA | NA (0.839) |
| Length | Acclim | 2 | 1.192 | 0.319 |
| | Sex | 1 | 4.012 | 0.0549 |
| | Acclim x Sex | 2 | NA | NA (0.419) |
| K | Acclim | 2 | 1.112 | 0.343 |
| | Sex | 1 | 0.285 | 0.598 |
| | Acclim x Sex | 2 | NA | NA (0.925) |
| RVM | Acclim | 2 | 8.015 | 0.00177 |
| | Sex | 1 | 0.148 | 0.703 |
| | Acclim x Sex | 2 | NA | NA (0.276) |

** The interaction term was not significant (NA) for any of the parameters, and thus, this term was taken out of the model and it was re-run.*
